# Transitioning to Remote Clinic Visits in a Smoking Cessation Trial During the COVID-19 Pandemic: Mixed Methods Evaluation

**DOI:** 10.2196/25541

**Published:** 2021-04-30

**Authors:** Martin Christopher Mahoney, Eunhee Park, Nicolas J Schlienz, CeCe Duerr, Larry W Hawk

**Affiliations:** 1 Roswell Park Cancer Institute Buffalo, NY United States; 2 School of Nursing University of Buffalo Buffalo, NY United States; 3 Department of Psychology University of Buffalo Buffalo, NY United States

**Keywords:** smoking, cessation, clinical trial, telehealth, COVID-19, coronavirus, telemedicine, conferencing, mixed methods, experience, patient

## Abstract

**Background:**

The pandemic of SARS-CoV-2, which causes COVID-19, has caused disruptions in ongoing clinical trials and is expected to accelerate interest in conducting research studies remotely.

**Objective:**

A quasi-experimental, mixed methods approach was used to examine the rates of visit completion as well as the opinions and experiences of participants enrolled in an ongoing clinical trial of smoking cessation who were required to change from in-person clinic visits to remote visits using video or telephone conferencing due to the COVID-19 pandemic.

**Methods:**

For quantitative comparisons, we used a quasi-experimental design, comparing a cohort of participants followed during the pandemic (n=23, COVID-19 cohort) to a comparable cohort of participants followed over a similar time period in the calendar years 2018 and 2019 (n=51, pre–COVID-19 cohort) to examine the rates of completion of scheduled visits and biospecimen collection. For the qualitative component, interviews were conducted with participants who experienced the transition from in-person to remote visits.

**Results:**

Participants in the COVID-19 cohort completed an average of 83.6% of remote clinic visits (95% CI 73.1%-91.2%), which was not significantly different than the in-person completion rate of 89.8% in the pre–COVID-19 cohort. Participants in the COVID-19 cohort returned an average of 93.2% (95% CI 83.5%-98.1%) of saliva specimens for remote clinic visits completed, which was not significantly different than the in-person saliva specimen completion rate of 100% in the pre–COVID-19 cohort. Two broad themes emerged from the qualitative data: (1) the benefits of remote visits and (2) the challenges of remote counseling compared to in-person counseling. Despite limited experience with telehealth prior to this transition, most participants expressed a willingness to engage in remote visits in the future.

**Conclusions:**

Even in the context of a rapid transition from in-person to remote visits necessitated by the COVID-19 pandemic, rates of visit completion and return of biospecimens remained high. Participants were generally accepting of the transition. Further research is needed to identify the optimal mix of in-person and remote visits beyond the pandemic context and to better understand how these changes may impact study outcomes.

**Trial Registration:**

ClinicalTrials.gov NCT03262662; https://clinicaltrials.gov/ct2/show/study/NCT03262662

## Introduction

Recent reviews have examined the growing interest in conducting remote trials for smoking cessation by incorporating existing applications for cell phones and other technology [1-3]. However, transitioning from the more traditional approach, in which study participants return for assessments at a research center, to a more decentralized design raises multiple implementation issues, including study recruitment and screening, completion of informed consent, follow-up assessments, biomarker collection, and medication adherence monitoring [1]. Although technology currently exists to support decentralized assessments, data collection, and other procedures, the processes of these transitions and their impact on participant and clinician experiences remains unclear [4-9].

The SARS-CoV-2 outbreak, which led to the COVID-19 pandemic, has caused disruptions in ongoing clinical trials and is expected to accelerate interest in conducting research studies remotely. Many trials were suspended for varying periods of time, while others were forced to transition from in-person to remote visits. In this context, the COVID-19 pandemic experience provided a unique opportunity to examine quantitative and qualitative data, albeit nonexperimentally, regarding the impact on relevant intermediate study outcomes such as completion of visits and return of biomarker specimens, as well as to consider participant perspectives on the transition and their preferences and recommendations for in-person versus remote visits.

This paper uses a quasi-experimental, mixed methods approach to examine the opinions and experiences of participants enrolled in an ongoing clinical trial of smoking cessation who were required to change from in-person clinic visits to remote visits using video or telephone conferencing due to the COVID-19 pandemic. The quantitative component allows for direct (albeit nonexperimental) comparisons among a group of participants on objective measures of effect, which is complemented by qualitative data regarding the experiences and opinions of research participants. For quantitative comparisons, we used a quasi-experimental design, comparing a cohort of participants followed during the pandemic to a comparable cohort of participants followed over a similar time period in the calendar years 2018 and 2019 to examine rates of completion of scheduled clinic visits and biospecimen collection. We tested whether rates of visit completion and saliva collection were lower for remote visits than for in-person visits. For the qualitative approach, interviews were conducted with a subset of participants who experienced the transition from in-person to remote visits.

## Methods

### Clinical Trial

Our ongoing randomized clinical trial examines the impact of an alternative varenicline dosing strategy to enhance rates of cessation among treatment-seeking adult cigarette smokers [10]. Following prescreening and completion of a baseline visit, eligible participants are randomized to one of two varenicline dosing regimens and scheduled for 6 clinic visits over a 2-month period, during which survey measures are completed, biospecimens are collected, and brief individual cessation counseling is delivered. Study outcomes include bio-verified continuous abstinence at end-of-treatment and at 6 months; the focus of this paper is on the active treatment phase of the study.

In March 2020, accruals to this trial were on target when the COVID-19 pandemic forced us to rapidly implement changes to our procedures. One of the primary changes involved a transition from in-person counseling to remote counseling via Zoom (Zoom Video Communications, Inc) or telephone calls (when Zoom was not feasible for the participant) for the 23 participants who had already begun the treatment phase but had not yet completed all clinic visits (ie, the COVID-19 cohort).

### Study Design

A mixed methods approach, based on a concurrent embedded framework, was used to explore the impact of transitioning to remote clinic visits in a smoking cessation trial during the COVID-19 pandemic. A quasi-experimental design provided quantitative data to compare clinic visit completion rates among the COVID-19 cohort to a comparable cohort of participants with clinic visits that were scheduled during a similar time period in the calendar years 2018 and 2019 (ie, the pre–COVID-19 cohort). Qualitative methods [11] were used to examine the experiences of in-person versus remote clinic visits completed via video or telephone conferencing among a subgroup of participants enrolled in an ongoing clinical trial of smoking cessation. Participants in both cohorts must have had at least one additional clinic visit remaining after having completed a clinic visit during the transitional time period (March-April 2020) or during a parallel interval in 2018 or 2019. Qualitative interviews were conducted with 15 of the 23 participants (65%) in the COVID-19 cohort.

A mixed methods design with a convergent approach was used to inform a more comprehensive understanding of the impact of transitioning from in-person to remote clinic visits by integrating both qualitative and quantitative data instead of only relying on one of these data sources. The quantitative data test the major outcomes of the quasi-experimental condition, while the qualitative data provide important insights about the process from people’s experiences [12,13].

### Study Participants

The parent research project (ClinicalTrials.gov NCT03262662) continues to enroll eligible treatment-seeking cigarette smokers between the ages of 18 and 70 years in a randomized clinical trial evaluating extended pre-quit varenicline pharmacotherapy for smoking cessation. Following intake and randomization, each clinical trial participant is scheduled to complete 6 in-person clinic visits (at weeks 0, 1, 3, 4, 6 and 8, respectively) as well as 2 follow-up appointments to assess long term abstinence. At each in-person clinic appointment, participants complete blood pressure and exhaled carbon monoxide (CO) assessments, fill out self-administered questionnaires, provide a saliva sample, receive brief behavioral counseling, and receive a supply of study medication.

#### COVID-19 Cohort

For the current investigation, we focused on a COVID-19 cohort, defined as participants (n=23) who had completed at least 1 in-person clinic visit in a 2-week transition window immediately before the PAUSE executive order was issued in New York State and had at least 1 of 6 clinic visits remaining (range: 1-5 remaining visits). In other words, these participants completed clinic visit #1, 2, 3, 4, or 5 during the 2-week transition period. The PAUSE executive order, issued in mid-March 2020, suspended 100% of nonessential business activities, including clinical research.

During the transition window clinic visit, participants were provided with their remaining supply of study medication and remote visit packets containing instructions, paper copies of self-administered questionnaires, behavioral counseling handouts, and at-home saliva sample collection kits, along with postage-paid envelopes for returning the surveys and specimens. Procedures for either telephone or Zoom meetings were also briefly reviewed.

For the remaining clinic visits, the questionnaires could be completed by following an email link to the surveys programmed in Research Electronic Data Capture (REDCap) or by filling out paper copies of the measures included in the remote visit packets. Participants received audio (telephone) or audiovisual (Zoom) instructions to guide saliva sample collection. To return the remote clinic visit packet to the study investigators, participants sealed their completed questionnaires and saliva specimen in a large padded envelope with pre-paid postage that could be picked up by their mail carrier.

#### Pre–COVID-19 Cohort

For comparative purposes, we retrospectively reviewed records to identify a comparator cohort of study participants who had completed an in-person clinic visit during the same 2-week time frame (March 18 to April 2) in the calendar year 2018 or 2019 and had 1 or more (range 1-5) remaining clinic visits scheduled (ie, the pre–COVID-19 cohort). Participants followed during 2018 and 2019 did not significantly differ with regard to demographic or tobacco use characteristics and were combined into a single pre–COVID-19 cohort (n=51).

### Measures

#### Baseline Variables

We examined participant age, sex, race/ethnicity, household income, highest level of education completed, Fagerstrom Test of Cigarette Dependence (FTCD) cigarettes per day at time of enrollment, and baseline exhaled carbon monoxide level across the COVID-19 and pre–COVID-19 cohorts. The FTCD is a 6-item self-reported scale used to assess the severity of dependence on cigarettes; the level of dependence is inversely correlated with the ability to successfully quit [14,15].

#### Dependent Variables

We focused on the percentage of clinic visits completed and the rates of biospecimen (saliva) collection after the last in-person visit for the COVID-19 cohort and after the comparable in-person visit for the pre–COVID-19 cohort.

### Data Analyses

This study relied upon a concurrent design in which quantitative and qualitative data collection and analyses were performed in parallel to yield complementary impressions of the impact of transitioning from in-person interviews and a desire to not burden participants to the extent that it would negatively affect the completion of long-term follow-up visits for participants still participating in the trial [13,16]. Consistent with a contiguous approach to data integration, the quantitative and qualitative results will be reported in different sections, followed by a synthesis of these findings in the *Discussion* section [16].

#### Quantitative Analyses

Descriptive statistics, *t* tests, and chi-square tests were used to compare means and categorical distributions by cohort; *P* values <.05 were considered significant. Analyses were conducted using SPSS, version 26 (IBM Corporation).

#### Qualitative Analyses

Persons in the COVID-19 cohort were contacted by telephone and invited to participate in a telephone-based structured interview to explore their experiences with transitioning from in-person to remote visits. Interviews were completed shortly after the final scheduled clinic visit (visit #6). Participants were informed verbally of the study aims and provided verbal informed consent; no additional compensation was provided. Interviews were completed by 2 trained research assistants using a structured interview guide. Items in the structured interview addressed how in-person counseling visits compared with remote visits, whether most remote visits were telephone calls or video sessions, desirable and undesirable features of remote visits, challenges encountered during remote visits, experiences with remote survey completion and saliva sample collection, privacy issues regarding in-person and remote visits, prior experience with telemedicine, preferences for remote versus in-person visits, and how changing from in-person to remote visits might have influenced the participant’s ability to stop smoking. The interviews lasted an average of 12:48 minutes (range 7:01-26:03), excluding informed consent. Interviews were completed with 15/21 persons (71%); 2 persons did not complete any remote visits and were not contacted, 4 persons declined to participate, and several telephone calls yielded no response for 2 participants. Initial interview audio transcripts were generated by the Zoom software. Individual transcripts were carefully reviewed by a coauthor (NJS), who made minor corrections when necessary to address errors or to improve clarity. This research was approved by the Institutional Review Board (IRB) at the University at Buffalo.

A qualitative descriptive analysis was conducted using a conventional content analysis [17], and ATLAS.ti software (ATLAS.ti Scientific Software Development GmbH) was used for the qualitative coding process. With an inductive approach, each transcript was thoroughly read and coded based on the unit of meanings [18]. Data from all participants were combined and analyzed to identify themes. After the initial coding process, the coding was discussed and reviewed by 2 coauthors (EP and MCM), then grouped and categorized under two main themes: (1) the benefits and (2) the challenges of remote counseling compared to in-person counseling. In addition, descriptive statistics were used to report the respondent percentages regarding the platform used (telephone only or video), preferred setting for clinic visits (on-site or remote), previous experiences with telemedicine, future recommendations for visit type, willingness to try telehealth, and the self-perceived impact of remote counseling on quitting success.

## Results

### Study Participants

Table 1 presents selected demographic, tobacco use, and study completion characteristics for the study participants in the COVID-19 and pre–COVID-19 cohorts (n=23 and 51, respectively). The cohorts were comparable on all demographic and tobacco use characteristics examined. Also, the cohorts did not differ with regard to loss to follow-up (1/23, 4%, vs 0/51, 0%) or withdrawals from the study (1/23, 4%, vs 5/51, 10%).

**Table 1:** Selected demographic, smoking, and visit attendance characteristics among smoking cessation clinical trial participants by study cohort.

Characteristic	Values		*P* value
	COVID-19 cohort^a^ (n=23)	Pre–COVID-19 cohort^b^ (n=51)	
Age (years), mean (SD)	53.8 (9.6)	54.8 (9.3)	.68
Female sex, n (%)	10 (44)	28 (55)	.36
Did not self-identify as White, n (%)	7 (30)	12 (24)	.53
Household income <US $50,000, n (%)	9 (39)	19 (37)	.48
High school degree or less, n (%)	9 (35)	15 (29)	.64
Baseline CPD^c^, mean (SD)	20.5 (8.4)	19.5 (7.4)	.60
Baseline CO^d^, mean (SD)	20.9 (12.1)	16.6 (18.8)	.32
FTCD^e^ score, mean (SD)	5.9 (1.8)	6.2 (1.7)	.52
Clinic visits during transition window^f^ (range 1-5), mean (SD)	2.8 (1.5)	2.9 (1.4)	.70
Lost to follow-up, n (%)	1 (4)	0 (0)	.13
Withdrew from study, n (%)	1 (4)	5 (10)	.43

^a^COVID-19 cohort: participants with mix of in-person and remote clinic visits due to the COVID-19 pandemic.

^b^Pre–COVID-19 cohort: participants with all in-person clinic visits scheduled during a comparable time period during the calendar years 2018 and 2019.

^c^CPD: cigarettes per day.

^d^CO: exhaled carbon monoxide at baseline visit.

^e^FTCD: Fagerstrom Test for Cigarette Dependence.

^f^Transition window: matching on-site visit during a similar calendar year period in 2018 and 2019.

### Quantitative Data

21 of 23 participants in the COVID-19 cohort (91%) completed at least 1 remote clinic visit, while 48 of 51 participants (94%) in the pre–COVID-19 cohort completed at least 1 subsequent in-person visit; withdrawals and loss to follow-up were counted as incomplete visits. As presented in Table 2, participants in the COVID-19 cohort completed an average of 83.6% of their remote clinic visits (95% CI 73.1%-91.2%), which was not significantly different than the in-person completion rate of 89.8% (95% CI 84.0%-94.1%) in the pre–COVID-19 cohort (Table 3). Participants in the COVID-19 cohort returned an average of 93.2% (95% CI 83.5%-98.1%) of saliva specimens for remote clinic visits completed, which was not significantly different from the in-person saliva specimen completion rate of 100% (95% CI 97.4%-100%) in the pre–COVID-19 cohort (see Tables 4 and 5).

When given the option of recommending either in-person or remote clinic visits for a future cessation trial, 11/15 interviewed participants (73%) expressed a preference for a hybrid approach, with 7 participants recommending that future clinic visits be scheduled as a mix of in-person and remote visits and 4 respondents indicating that participants should be permitted to select their own schedule of clinic visits; 3 participants expressed a preference for all in-person visits, and 1 indicated no preference. Also, 13/15 participants (87%) reported that the change from in-person to remote visits had no impact on their ability to stop smoking; 1 participant reported a positive impact, and 1 reported a “potentially negative” impact from this change.

**Table 2:** Clinic visit attendance among smoking cessation clinical trial participants for the COVID-19 cohort (n=23).

Last in-person visit	n	Remote clinic visit completion, n (%)^a^						Remote visit attendance (%)
		Visit #2	Visit #3	Visit #4	Visit #5	Visit #6		
Visit #1	5	4 (80)	4 (80)	3 (60)	3 (60)	3 (60)	68	
Visit #2	6	N/A^b^	5 (83)	5 (83)	4 (67)	4 (67)	75	
Visit #3	4	N/A	N/A	4 (100)	3 (75)	3 (75)	83	
Visit #4	4	N/A	N/A	N/A	4 (100)	4 (100)	100	
Visit #5	4	N/A	N/A	N/A	N/A	4 (100)	100	
Total	23	4 (80)	9 (82)	12 (80)	14 (74)	18 (78)	83.6^c^	

^a^Percentages in the Total row are calculated based on the sum of visits in each column.

^b^N/A: not applicable.

^c^95% CI 73.1%-91.2%.

**Table 3:** Clinic visit attendance among smoking cessation clinical trial participants by last in-person visit for the pre–COVID-19 cohort (n=51).

Matching in-person visit^a^	n	On-site clinic visit completion, n (%)^b^						On-site visit attendance (%)
		Visit #2	Visit #3	Visit #4	Visit #5	Visit #6		
Visit #1	11	11 (100)	11 (10)	10 (91)	10 (91)	8 (73)	91	
Visit #2	9	N/A^c^	7 (78)	7 (78)	7 (78)	7 (78)	78	
Visit #3	14	N/A	N/A	14 (100)	14 (100)	13 (93)	98	
Visit #4	7	N/A	N/A	N/A	6 (86)	6 (86)	86	
Visit #5	10	N/A	N/A	N/A	N/A	10 (100)	100	
Total	51	11 (100)	18 (90)	31 (91)	37 (93)	44 (86)	89.8^d^	

^a^Matching: on-site visit during a similar period in 2018 and 2019.

^b^Percentages in the Total row are calculated based on the sum of visits in each column.

^c^N/A: not applicable.

^d^95% CI 84.0%-94.1%.

**Table 4:** Saliva sample collection rates among smoking cessation clinical trial participants by last in-person visit for the COVID-19 cohort (n=23).

Last in-person visit	n	Remote saliva sample collection, samples/visits completed (%)						Saliva sample collection rate (%)
		Visit #2	Visit #3	Visit #4	Visit #5	Visit #6		
Visit #1	5	3/4 (75)	3/4 (75)	3/3 (100)	3/3 (100)	3/3 (100)	88	
Visit #2	6	N/A^a^	5/6 (83)	5/6 (83)	4/4 (100)	4/4 (100)	90	
Visit #3	4	N/A	N/A	4/4 (100)	3/3 (100)	3/3 (100)	100	
Visit #4	4	N/A	N/A	N/A	4/4	4/4	100	
Visit #5	4	N/A	N/A	N/A	N/A	4/4	100	
Total	23	3/4 (75)	8/10 (80)	12/13 (92)	14/14 (100)	18/18 (100)	93.2^b^	

^a^N/A: not applicable.

^b^95% CI 83.5%-98.1%.

**Table 5:** Saliva sample collection rates among smoking cessation clinical trial participants by last in-person visit in the pre–COVID-19 cohort (n=51).

Matching in-person visit^a^	n	On-site saliva sample collection, samples/visits completed (%)						Saliva sample collection rate (%)
		Visit #2	Visit #3	Visit #4	Visit #5	Visit #6		
Visit #1	11	11/11 (100)	11/11 (100)	10/10 (100)	10/10 (100)	8/8 (100)	100	
Visit #2	9	N/A^b^	7/7 (100)	7/7 (100)	7/7 (100)	7/7 (100)	100	
Visit #3	14	N/A	N/A	14/14 (100)	14/14 (100)	13/13 (100)	100	
Visit #4	7	N/A	N/A	N/A	6/6 (100)	6/6 (100)	100	
Visit #5	10	N/A	N/A	N/A	N/A	10/10 (100)	100	
Total	51	11/11 (100)	18/18 (100)	31/31 (100)	37/37 (100)	44/44 (100)	100^c^	

^a^Matching: on-site visit during a similar period in 2018 and 2019.

^b^N/A: not applicable.

^c^95% CI 97.4%-100%.

### Qualitative Data

Two broad themes emerged from the qualitative data: (1) the benefits of remote visits and (2) the challenges of remote counseling compared to in-person counseling. As presented in Table 6, the benefits of remote clinic visits included not having to travel to in-person appointments (noted by 11 participants), feeling more comfortable in their homes (7 participants), generally easier and more convenient (5 participants), feeling safer staying at home during the COVID-19 pandemic (3 participants) and a greater level of privacy when completing the clinic visits from their homes (3 participants). Challenges associated with the remote visits included less personal interaction and support (noted by 6 participants), challenges with technology (5 participants), less accountability to complete assignments (5 participants), less confidence in their ability to quit smoking (4 participants) and distractions at home (2 participants).

Among the 15 participants interviewed, 8 (53%) reported that the majority of their remote counseling visits were video calls (audio and video communication); a strong preference was expressed for video calls (noted by 7/9 participants, 78%, who completed at least one video call and at least one telephone call), as video sessions were viewed as more personal and interactive (6 participants completed only video calls or only telephone calls). Few participants (2/15, 13%) reported any previous experience with video or audio calls from their health care providers (eg, telehealth visits or virtual visits). Following the experience of these remote visits, 13/15 participants (87%) indicated a willingness to engage in telehealth visits in the future.

**Table 6:** Dominant themes and subthemes based on structured interviews with smoking cessation clinical trial participants (n=15) who completed both in-person and remote clinic visits.

Theme and subthemes			n (%)		Illustrative quotes
**Benefits of remote clinic visits**					
	No travel required	11 (73)		“I guess it saved a little time, didn't have to drive there. But, you know, so it's basically a little more time-consuming to actually go there.” “Just saves the trip. Don't have to worry about trying to find a place to park or anything.”	
	Safer	3 (20)		“With COVID-19 being prevalent, it felt safer…” “I'm just saying on my behalf, it was better for me to be at home, especially during the pandemic.”	
	Generally easier/more convenient	5 (33)		“Of course you got to take the time off to get there.” “I didn’t have to wait to be seen or anything, when the call came I accepted the call and we went from there.” “It’s just far more easier and convenient.”	
	More comfortable	7 (47)		“You’re much more comfortable in your own environments. That’s the way I felt. It’s not that staged clinical setting…there’s comfort in your own home, your own environment is very comforting and calming.” “You’re a little bit more comfortable with doing things because you know, you’re at home.”	
	Greater privacy at home	3 (20)		“Obviously it's more private being at home because you can control, even though I have no issues with privacy when I came to the office.” “I live alone so nobody else was there. Nobody hearing my thoughts and it felt as if I could express myself a little better, for me.”	
**Challenges of remote clinic visits**					
	Less personal/less supportive	6 (40)		“I think connecting with the counselor was easier in person.” “It was more personal being there (in-person) compared to doing a Zoom meeting…” “You lose that factor of like meeting staff and other people there, that was kind of nice you know. Like knowing there’s something behind the program you are in.” “Sometimes it's easier just explaining things when you're there in person and you can show what kind of stuff.”	
	Distractions at home	2 (13)		“I have 3 dogs that are really loud so I have to make sure they are all locked up” “People at home could, you know, hear my phone call…”	
	Technology challenges	5 (33)		“Zoom is new to me. Video conferencing is new to me….” “I like it a lot better in person because I’m not a technology…”	
	Less self-confidence in ability to quit	4 (27)		“I’m at home and I might pick up a cigarette.” “Probably a little less confident, but I did it. It was like, it's tough when you don't have friends or doctors and people that were rooting you on.”	
	Less accountability	5 (33)		“I was preparing a little bit more [for office visits] than I do when just the phone rings.” “Driving there, part of me quitting is me thinking about quitting and I have spent half an hour to get there you know, and block out a portion of my day, hey, I can't wait to quit because when I do I won't have to do this anymore.” “For me personally, I tended to work on the homework in between sessions. On days prior to my sessions when it was in-person. At-home visits, I just didn't feel the accountability to do that at the at-home visits.”	

## Discussion

The quasi-experimental component identified no differences between subcohorts in the proportion of participants lost to follow-up or withdrawal from the study. Participants in the COVID-19 cohort completed an average of 83.6% (95% CI 73.1%-91.2%) of scheduled remote clinic visits and returned an average of 93.2% (95% CI 83.5%-98.1%) of saliva specimens, which was comparable to the in-person clinic visit completion rate of 89.8% (95% CI 84.0%-94.1%) and the in-person saliva specimen completion rate of 100% (95% CI 97.4.0%-100%) in the pre-COVID-19 cohort. The qualitative findings suggested two broad themes: (1) the benefits of remote visits, such as convenience, comfort and safety, and (2) the challenges of remote visits, such as less personal interaction with the study staff, struggles with technology, less accountability to complete assignments, and decreased confidence in their ability to quit smoking. There was a strong preference expressed for video calls over telephone calls, as video sessions were viewed as more personal and interactive. Finally, while few participants reported any previous experience with video or audio calls with their health care providers, nearly all participants indicated a willingness to engage in telehealth visits in the future.

The ongoing COVID-19 pandemic has heightened interest in remote, decentralized clinical trials. Dahne and colleagues [1] recently summarized the use of emerging technologies to support the remote implementation of smoking cessation trials, and our study employed several such tools. Their review described ongoing shifts, including use of Facebook or other social media platforms, to support study recruitment. With IRB approval, participants can give consent remotely; for example, REDCap provides the ability to complete the consent process electronically, along with audio or video calls with members of the research team to address questions. Survey instruments can be completed through email or SMS text message links sent to participants [1]. In this study, we used REDCap to send participants links to surveys, including safety monitoring, to be completed electronically as a component of these remote visits. Studies of smoking cessation have typically relied upon biomarker collection at in-person visits to validate smoking status. However, saliva specimens can be successfully collected remotely and mailed back to investigators. Also, it is possible to collect CO measurements using devices connected to smartphones (eg, the iCO Smokerlyzer monitor [19]). During biospecimen collection, video calls or facial recognition software can be used to verify participant identify, and incentives can be credited virtually to a ClinCard account. In this study, we observed specimen collection during most visits via Zoom; remuneration was not credited to a participant’s account until the specimen was received. Adherence to study medication can be assessed remotely via saliva specimens or using the Medication Event Monitoring System (MEMS), which records pill container openings. However, direct observation of medication self-administration, along with remote assessments of weight, height, and blood pressure, is potentially more intrusive and introduces several complexities [1]. In this study, we conducted remote medication accountability assessments; however, to make remote visits more feasible, we did not require participants to send back pill containers, and we dropped a number of secondary assessments (ie, weight, blood pressure, and CO) from the study.

In March 2020, the US Food and Drug Administration offered recommendations for ongoing and planned clinical trials [20], including “alternatives to in-person safety assessment (eg, phone contact, virtual visits, alternative locations for assessment), direct-to-patient investigational product delivery, the collection of efficacy endpoint data, replacing on-site monitoring with decentralized or remote monitoring, and additional safety monitoring of trial participants if the trial is halted or treatment is discontinued.” Consistent with these recommendations, we used the final in-person visit to provide participants in our clinical trial with the materials and resources to continue visits remotely. Importantly, the implications of these modifications remain unclear. Thus, we examined quantitative (rates of remote visit completion and rates of saliva specimen collection) and qualitative aspects of the transition to remote visits. To our knowledge, this study is among the first to explore participants’ experiences and the impact of such transitions in a smoking cessation trial during the COVID-19 pandemic.

The findings for visit completion were generally encouraging; we observed robust and sustained rates of completion for scheduled clinic visits after the migration to a remote platform of telephone and/or Zoom calls (83.6%) as a result of the COVID-19 pandemic. Importantly, the completion rate was not significantly lower than that of a historical comparison group, namely the pre–COVID-19 cohort comprising participants from the calendar years 2018 and 2019 who only experienced in-person visits (89.8%). Although the comparison is quasi-experimental, the two cohorts were comparable on all baseline participant and smoking characteristics, ruling out a variety of potential confounds. However, there is one critical confound between groups: participants in the COVID-19 cohort were also faced with the unique circumstances of the pandemic.

It is important to note that we were not powered to detect small group differences in visit completion rates. Conversely, the transition from in-person to remote visits was made quite rapidly and may represent a “worst case scenario.” We would anticipate that remote visit completion rates would be even better if participants were preinformed of the remote visits and as a result of further refinement and standardization of our procedures. Regarding remote visits more generally, it is important to note that none of the current participants anticipated the possibility of remote visits upfront. Visit completion rates may be even higher in trials that are specifically designed to be remote [21].

The second goal of this research was to understand the opinions and experiences of clinical trial participants who were required to change from in-person to remote visits after completing at least one in-person clinic visit. Interviews with 15 such participants suggested a mix of benefits and challenges for remote visits. Benefits included not having to travel to on-site appointments, avoiding potential exposure to COVID-19, and the general convenience of staying at home. Challenges of remote visits involved the less personal nature of interactions with project staff, technology challenges, and less accountability. Taken together, these data provide suggestions for more satisfying participant experiences in future intervention trials. For example, planning sufficient time and pre-educational sessions to deliver technology support may be necessary for successful remote visits via telephone or video. In addition, sending reminder calls or SMS text messages in advance of scheduled video or teleconferences seems to enhance participants’ accountability. Several participants in our study also commented on less privacy and more distractions at home, while others felt that privacy was greater at home. Few participants (2/15, 13%) who were required to transition to remote visits reported familiarity with virtual visits or telehealth at the time of migration to a remote platform. However, following their experience in this study, a large percentage (13/15, 87%) reported a willingness to participate with telehealth visits either via telephone or video in the future. After the experience of both in-person and remote visits, most participants recommended a mix of visit types or choosing from both options going forward. Studies comparing use of video counseling to telephone counseling have focused on specific groups of smokers (eg, women with HIV, Korean American women, rural residents in Kansas, rural residents in Australia) [6-8], limiting generalizability. Nonetheless, it seems both reasonable and practical to use video and telephone platforms to deliver counseling support to smokers interested in quitting.

Participants in our clinical trial were already familiar with electronic data capture (REDCap) for completing many study measures; therefore, the transition to remote visits may have had less of an impact on that component. We did lose the ability to assess exhaled breath CO; however, we successfully retained our primary bioverification measure (salivary cotinine), as 93% of all remote visits were accompanied by the participant sending back their saliva sample.

Adherence, measured as time spent in treatment, for face-to-face versus a mix of face-to-face and web visits was studied among 292 persons enrolled in a 10-session smoking cessation trial conducted in the Netherlands [5]. Adherence was similar in the 2 groups; however, the blended group demonstrated more treatment time spent during face-to-face visits compared to web visits; the only factor that predicted increased adherence was older age (*R*^2^=0.047). These authors concluded that in-person visits compensated for weaknesses of the web visits, which was also noted in qualitative research among smokers in this trial [22]. Our study assessed adherence based on completion of scheduled visits and did not track duration of visits. Finally, we observed that 93% of the remote visits in the COVID-19 cohort were accompanied by returned saliva specimens, suggesting that saliva specimen collection for bioverification of cessation or assessing study drug levels is feasible.

Limitations of this study include the modest size of the cohort that transitioned to remote clinic visits, limited power to examine differences in rates of completion by individual visits or among specific subgroups of participants, and the inability to assess nonrespondent bias. Also, the duration of the interviews with participants who completed remote clinical visits was generally shorter than what is typical for qualitative studies; however, this was reflective of the focused nature of the interview guide and a desire to not burden respondents to an extent that would negatively affect the completion of long-term follow-up visits for participants still in the trial. These modest limitations are offset by the unique strengths of our mixed methods approach, which supported an examination of opinions and experiences of participants enrolled in an ongoing clinical trial of smoking cessation who were required to change from in-person clinic visits to remote visits and the quasi-experimental design comparing rates of visit completion to a comparable group of participants followed over a similar time period in calendar years 2018 and 2019.

The experience of the COVID-19 pandemic has forced many investigators to more rapidly use and incorporate mobile health and technology applications into their ongoing clinical trials. This study suggests that participants in our smoking cessation trial were successfully transitioned from in-person to remote clinic visits based on high rates of visit and biospecimen completion and general satisfaction with the experience.

These unique analyses provide valuable information on the experiences and perspectives of participants enrolled in a clinical trial of smoking cessation who were transitioned from in-person to remote clinic visits due to the COVID-19 pandemic. This synthesis of qualitative and quantitative data support a successful transition process from in-person to remote visits for this smoking cessation clinical trial, as Dahne and colleagues [1] suggested. As such, this study also provides some initial direction for the design of future clinical trials, given the robust rates of completion for remote visits.

In summary, our findings indicate strong acceptability among participants in this clinical trial for completing clinic visits remotely after starting initially with in-person visits and suggest the potential importance of considering individual preferences with regard to potentially transitioning to remote visits. These study findings are consistent with promising results of studies in which technology was used to support remote delivery of smoking cessation treatment [3,6-8,23]. However, this study extends the possibilities to use of mobile and virtual modes for successful smoking cessation trial implementation, including assessment and data collection in the context of a pandemic. Furthermore, the mixed methods approach provides a more in-depth understanding of the potential impact on visit completion rates and experiences regarding the transition process among study participants who were not anticipating such changes. However, the optimal mix of in-person and remote visits remains undetermined, as does our understanding of how these changes may impact study outcomes. Finally, the successful transition of these clinical trial participants from in-person to remote visits was likely supported by several factors, including the development of rapport through in-person visits, provision of detailed instructions, the availability of both telephone or video platforms and the availability of support to trouble shoot technical issues, as well as strong motivation by participants to quit smoking.

## Abbreviations

CO: carbon monoxide
FTCD: Fagerstrom Test of Cigarette Dependence
IRB: Institutional Review Board
MEMS: Medication Event Monitoring System
REDCap: Research Electronic Data Capture
